# Association between frailty and postherpetic neuralgia in the older adult with herpes zoster

**DOI:** 10.3389/fpubh.2025.1511898

**Published:** 2025-02-12

**Authors:** Yunyan Shen, Ping Lin

**Affiliations:** ^1^The Fourth School of Clinical Medicine, Zhejiang University of Traditional Chinese Medicine, Hangzhou, Zhejiang, China; ^2^Department of Geriatrics, Hangzhou Third People's Hospital, Hangzhou, Zhejiang, China

**Keywords:** frailty, herpes zoster, postherpetic neuralgia, the older adult, pain

## Abstract

**Introduction:**

Chronic pain is closely related to frailty.

**Methods:**

A total of 300 older adult patients diagnosed with HZ were selected and admitted to our hospital in 2024. A basic data questionnaire gathered baseline information, and the FRAIL scale was employed to evaluate their initial frailty status. Patients who were non-frail at baseline were followed up for 3 months. They were categorized into the PHN group and the non-PHN group based on the occurrence of neuropathic pain during follow-up. Diverse scales were employed to evaluate frailty, nutritional status, anxiety, and depression among participants. The Chi-square or Kruskal-Wallis tests were employed to compare the two groups. The logistic regression model was undertaken to explore the impact of PHN on frailty.

**Results:**

Among the 300 older adult patients with HZ who satisfied the inclusion criteria, follow-up data were collected from 215 patients, comprising 85 cases in the PHN group and 130 cases in the non-PHN group. A prospective analysis of the 215 baseline non-frail patients showed that the incidence of frailty in older HZ patients was 14.9%. Univariate analysis revealed that the distributions of lesion site, lesion area, depression status, anxiety status, COPD, and nutritional score were statistically significant between the two groups (χ^2^ = 6.127, 4.846, 13.316, 12.967, 6.234, *H* = 2.592, *P* < 0.05). Nevertheless, the distributions of age, gender, marital status, education level, hypertension, and diabetes were not statistically significant (*P* > 0.05). Binary logistic regression analysis indicated that, after comprehensive adjustment for age, gender, lesion site, lesion area, depression status, anxiety status, COPD, and nutritional score, patients with PHN exhibited an higher risk of frailty compared to the non-PHN cohort (22 cases, OR = 3.279, 95% CI = 1.327–8.105; *P* = 0.010).

**Conclusion:**

Postherpetic neuralgia increases the risk of frailty and is a significant factor influencing the progression of frailty in the older adult.

## 1 Introduction

Frailty is a common geriatric syndrome characterized by diminished homeostatic reserve and recovery capability of the organism (2). Frailty in older adult persons elevates the risk of negative health outcomes, including falls, hospitalization, and mortality, attributable to diminished capacity to manage internal and external stressors and compromised recovery mechanisms. Thus, preventing or postponing the development of frailty is crucial for improving the quality of life in the older adult (3).

Previous studies have revealed a relationship between chronic pain and frailty (4–7), with the adverse health consequences of chronic pain contributing to the development of frailty. Research (8) has confirmed that in patients with osteoarthritis, pain increases the risk of frailty and can be used as a risk factor to increase the incidence of frailty (9, 10). Postherpetic neuralgia (PHN), as a typical chronic pain, has a high prevalence in the older adult population (11). However, its association with the emergence of frailty has not been revealed. Research indicates that sleep disorders, malnutrition, and negative psychological conditions resulting from PHN increase the risk of frailty in the older adult (12–15), suggesting a higher prevalence of frailty among individuals with PHN. PHN may play a significant role in the onset of frailty. Additionally, PHN can be alleviated through appropriate clinical interventions, which could operate as a modifiable factor to improve or even reverse frailty (16, 17). Investigating the frailty status of older adult patients with PHN and analyzing the impact of PHN on frailty progression in the older adult may enhance therapies for PHN, perhaps preventing, delaying, or even reversing the frailty process in older adults.

This study investigated the frequency of frailty in older adult patients with herpes zoster (HZ). We performed a prospective study of frailty incidence between older adult individuals with PHN and those without PHN. We explored the impact of PHN on the development of frailty in this population. These findings aim to raise awareness among healthcare professionals about the importance of effectively managing PHN, while also contributing to the prevention or delay of frailty progression in the older adult.

## 2 Materials and methods

### 2.1 Participants

This investigation is part of a frailty survey of hospitalized older adult patients with HZ in 2024. Participants were recruited from inpatients aged 65 and older at Hangzhou Third People's Hospital.

Inclusion criteria: (1) age ≥ 65 years; (2) diagnosed with HZ requiring hospitalization; (3) clear consciousness, ability to discern pain, and no communication barriers; (4) voluntary participation in the survey study. Exclusion criteria: those with concurrent serious mental diseases and cognitive problems who are incapable of effective communication. The Ethical Review Committee of the Third People's Hospital of Hangzhou approved this study (2024KA016). All subjects submitted written informed consent prior to their inclusion in the study.

### 2.2 Data and materials

The data collection employed the following survey instruments: the General Data Statistics Form, the FRAIL Scale, the Nutritional Risk Screening Tool for Hospitalized Patients (NRS 2002), the Self-Rating Anxiety Scale (SAS), the Geriatric Depression Scale (GDS-15), and the Numeric Rating Scale (NRS) for pain severity. Follow-up evaluations were performed either through telephone or outpatient appointments 3 months later.

### 2.3 Research instruments

#### 2.3.1 General data statistics form

A self-constructed general information questionnaire was employed, encompassing essential factors such as gender, age, educational attainment, marital status, smoking and alcohol consumption behaviors, chronic disease history, height, weight, lesion location, lesion size, and analgesic types utilized.

#### 2.3.2 The FRAIL scale

This scale was proposed in 2008 and is suitable for screening of the clinically age-deficient population (18, 19). The scale consists of the following five items: fatigue, difficulty in climbing one flight of stairs independently, difficulty in walking 100 meters independently on level ground, coexistence of five or more diseases, and weight loss. Each item is scored 1 point and a total score of 3 is considered frailty.

#### 2.3.3 The nutritional risk screening tool for hospitalized patients (NRS 2002)

The scale evaluates three primary factors: illness state, compromised nutritional status, and age; a score of 3 signifies malnutrition and the necessity for nutritional intervention.

#### 2.3.4 The self-rating anxiety scale (SAS)

The scale has 20 items and serves as a clinical instrument for evaluating patients' subjective experiences of chronic pain. The SAS standardized score has a cut-off value of 50, with scores of 50–59 indicating mild anxiety, 60–69 indicating moderate anxiety, and 70 or above indicating severe anxiety.

#### 2.3.5 The geriatric depression scale (GDS-15)

The scale comprises 15 items, classified into four factors: dissatisfaction, apathy and anxiety, loss of hope, memory impairment, and diminished social engagement. The scoring process is straightforward, and researchers and psychologists have extensively acknowledged its reliability and validity, rendering it an invaluable instrument for evaluating depression symptoms in the older adult. A score of 5 or above suggests the possible existence of depressive symptoms. A score of 5–8 signifies mild depression, 9–11 denotes moderate depression, and 12–15 reflects severe depression.

#### 2.3.6 The numeric rating scale (NRS)

The scale is extensively utilized for pain evaluation. The scale has 10 consecutive integers graded from 0 to 10, where 0 indicates no pain, 1–3 denotes mild pain, 4–6 signifies moderate pain, and 7–10 represents severe pain.

### 2.4 Statistical analysis

Statistical analyses were conducted using IBM SPSS Statistics, version 25.0 (IBM Corporation, China). The chi-square test was employed to compare the absolute (*n*) and relative (%) frequency distributions of categorical variables. The two groups were analyzed using the chi-square test or the Kruskal-Wallis test. A binary logistic regression model was applied for the analysis of influencing factors. *P* < 0.05 was considered statistically significant.

## 3 Results

**Figure 1:**
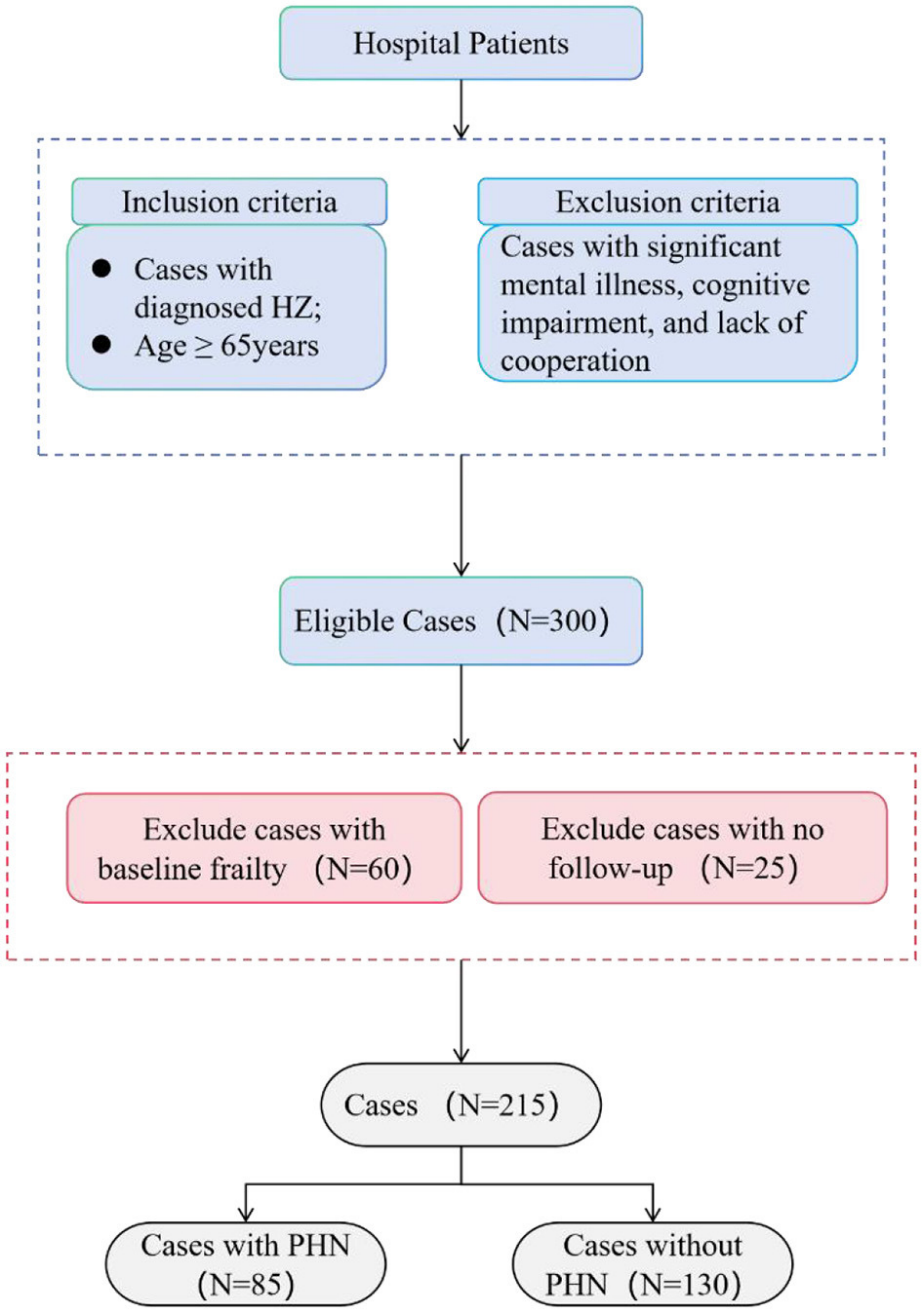
Flowchart depicting case selection in the study.

Three hundred individuals with HZ were enrolled in the trial after applying the inclusion criteria. After the 3-month follow-up period, we removed data from patients who had lost their follow-up appointments or showed frailty at baseline. In conclusion, the analysis encompassed data from 215 HZ patients aged 65. The average age of the complete sample was 72.82 ± 6.03 years (see Figure 1).

### 3.1 Comparative analysis of general information between the PHN group and the non-PHN group after the follow-up period

Table 1 delineates the characteristics of HZ patients who reported PHN (*n* = 85) and those who did not report PHN (*n* = 130), including age, gender, marital status, body mass index (BMI), education level, and lesion regions. In the cohort of PHN patients, 22 individuals (25.9%) indicated experiencing head or facial neuralgia, 49 individuals (57.6%) reported trunk neuralgia, and 14 individuals (16.5%) reported limb neuralgia. Most PHN patients were female, including 50 individuals, or 58.8%. PHN patients exhibited bigger lesion areas and elevated nutritional ratings than HZ patients without PHN. The incidence of anxiety and depression was elevated among PHN patients. PHN patients exhibited a greater prevalence of chronic obstructive pulmonary disease (COPD) concerning comorbidities. Furthermore, PHN patients utilized a broader range of analgesics.

**Table 1:** Anthropometric, demographic, and medical characteristics of the study participants.

**Variable**	**HZ without PHN**	**HZ with PHN**	**χ^2^ or *H* value**	***P*-value**
Age	72.44 ± 5.88	73.41 ± 6.26	1.157	0.248
Sex [*n* (%)]			1.608	0.205
Female [*n* (%)]	65 (50)	50 (58.8)		
Male	65 (50)	35 (41.2)		
Marriage [*n* (%)]			1.284	0.529
Married	116 (89.2)	72 (84.7)		
Divorced	1 (0.8)	1 (1.2)		
Widowed	13 (10.0)	12 (14.1)		
Education level [*n* (%)]	1.765	0.184		
Fewer than 6 years	64 (49.2)	34 (40.0)		
More than 6 years	66 (50.8)	51 (60.0)		
*BMI*	23.32 ± 2.82	23.68 ± 3.40	0.852	0.395
Skin lesion site [*n* (%)]			6.127	0.047
Head or face	51 (39.2)	22 (25.9)		
Trunk	53 (40.8)	49 (57.6)		
Limbs	26 (20.0)	14 (16.5)		
Skin lesion size [*n* (%)]	4.846	0.028		
<1%	56 (43.1)	24 (28.2)		
≥1%	74 (56.9)	61 (71.8)		
Depressed [*n* (%)]			13.316	<0.001
Yes	12 (9.2)	24 (28.2)		
No	118 (90.8)	61 (71.8)		
Anxiety [*n* (%)]	12.967	<0.001		
Yes	28 (21.5)	38 (44.7)		
No	102 (78.5)	47 (55.3)		
Nutritional scores [*n* (%)]	1.15 ± 1.03	1.58 ± 1.29	2.592	0.010
Hypertension [*n* (%)]			1.802	0.180
Yes	69 (53.1)	53 (62.4)		
No	61 (46.9)	32 (37.6)		
Diabetes [*n* (%)]			0.404	0.525
Yes	23 (17.7)	18 (21.2)		
No	107 (82.3)	67 (78.8)		
COPD [*n* (%)]			6.234	0.013
Yes	0 (0.0)	4 (4.7)		
No	130 (100.0)	81 (95.3)		

As shown in Table 2, diabetes mellitus and hypertension were the most prevalent chronic disorders in the PHN group in this study (*n* = 13 and 6, respectively).

**Table 2:** Frequency of chronic disorders in the PHN group in this study.

**Chronic diseases**	**Frequency**
Hypertension	53
Diabetes mellitus	18
COPD	4
Herniated lumbar disk	5
Malignant neoplasms	6
Coronary artery disease	9
Hypertriglyceridemia	12
Cerebral infarction	9
Benign prostatic hyperplasia	4

**Figure 2:**
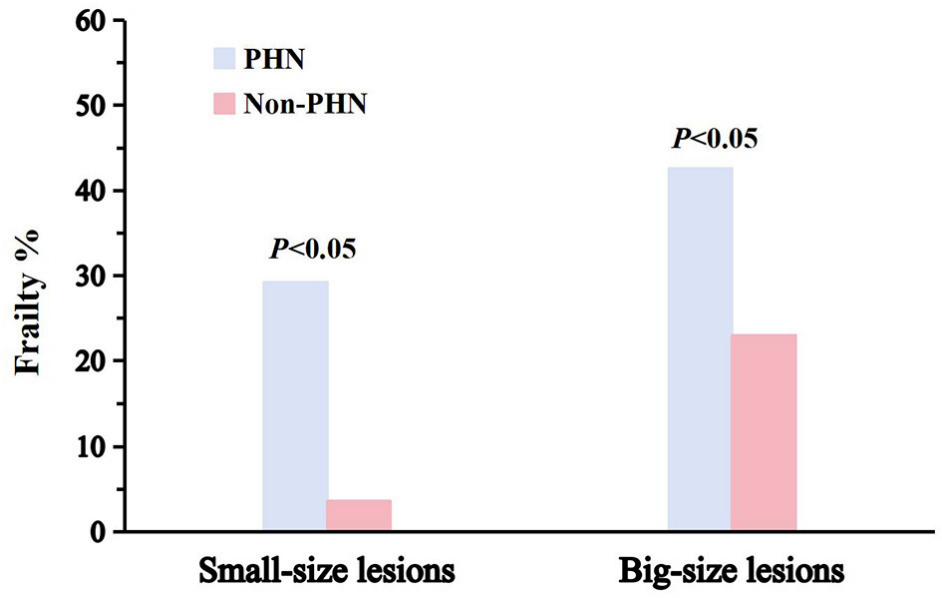
Prevalence of frailty in patients with PHN. The initial column denotes the prevalence of frailty in patients with PHN; the adjacent box signifies the prevalence of frailty in patients without PHN. The incidence of impairment for two lesion dimensions is shown.

Figure 2 shows the prevalence of frailty in PHN patients with different lesion sizes. As can be seen, when the two groups were compared, a significantly higher prevalence of frailty was found in patients with PHN across all lesion sizes (small-size lesions, 29.2% vs. 3.6%; big-size lesions, 42.6% vs. 23.0%; *P* < 0.05 for patients with PHN vs. patients with HZ without PHN).

Logistic regression analysis after fully adjusting for age, sex, nutrition, anxiety, depression, site of onset, area of skin lesions, and COPD found an association between PHN and the onset of frailty at follow-up (OR = 3.279, OR 95% CI 1.327–8.105, *P* = 0.010; Table 3).

**Table 3:** Relationship between PHN and frailty after the study across the complete follow-up population.

**Variable**	**Group**	**Unadjusted**			Adjusted^*^		
		**OR**	**OR 95% CI**	***P***	**OR**	**OR 95% CI**	***P***
PHN	No^#^						
*n* (%)	Yes	4.190	1.869–9.394	0.001	3.279	1.327–8.105	0.010

* Adjusted for age, gender, nutrition, anxiety, depression, site of onset, skin lesion size, and COPD;

# Control group.

## 4 Discussion

This prospective study revealed a significant increase in the incidence of frailty of frailty among older adult patients with PHN compared to those without, after controlling for confounding variables. Our study indicates that PHN is a risk factor for the onset of frailty in the older adult, particularly in PHN patients with large skin lesions, who are at an elevated risk of frailty.

This study found that the prevalence of PHN in HZ patients was 28.3%, consistent with earlier research findings (20, 21). PHN may last for months or even years and is a chronic pain disorder that afflicts older persons. In a rapidly aging society, formulating effective prevention strategies for frailty in the older adult is a critical concern because of the detrimental effects of frailty on health (22). Chronic pain as a risk factor for the development of frailty (23). A meta-analysis indicates that, after an average follow-up of 5.8 years, older adults with chronic pain were nearly twice as likely to develop frailty compared to those without chronic pain (24). The current investigation found that the prevalence of frailty in HZ patients after follow-up was 14.9%, exceeding the 6.9% prevalence in the community-based senior population, as reported by Fried et al. (25). Veronese et al. (8), in a prospective study with a mean follow-up of 4.4 years involving older patients with osteoarthritis (OA), discovered that OA-related pain affected the relationship between OA and frailty. Bindawas et al. (5) indicated in longitudinal research that the status of knee pain correlated with an increased incidence of frailty with time. Nevertheless, our research diverges from prior investigations. We investigated the relationship between PHN and the onset of frailty. We sought to elucidate the impact of PHN on the prevalence of frailty by stratifying the sample based on the size of skin lesions, categorizing them as small or large. In addition, we found a high prevalence of diabetes and hypertension in patients with frailty, and debilitation was associated with pain levels, nutrition, anxiety, and chronic obstructive pulmonary disease. These result in increased restrictions on daily functional activities and a greater likelihood of polypharmacy in older adults, conditions closely linked to the onset of frailty (26, 27). PHN elevates the incidence of frailty in older adult individuals, indicating that those with PHN constitute a high-risk demographic.

The link between PHN and frailty can be elucidated through a variety of interrelated mechanisms. Patients with PHN exhibit fatigue and lower levels of physical activity, perhaps resulting in impaired skeletal muscle function and heightened vulnerability to challenges in everyday tasks. Furthermore, patients with PHN suffer from detrimental health events, including malnutrition, sleep disorders, negative psychological states, and multimorbidity coexistence, all of which are associated with the onset of frailty. Hunt et al. (28) propose that older persons suffering from chronic pain, sleep deprivation, and inadequate nutrition may have a reduction in physiological reserve, hence heightening the risk of falls and cognitive impairment. Older adults with PHN patients are more prone to a sedentary lifestyle due to diminished immunity and restricted physical activity, with treatment guidelines advising extensive rest. Sedentary behavior contributes to muscle mass and strength deterioration, leading to skeletal sarcopenia and facilitating the emergence of frailty (1). These findings underscore the necessity of including frailty in therapeutic strategies for chronic pain management in patients with PHN, along with the need for customized exercise regimens and dietary therapies for those with compromised PHN. Studies have shown (29) that the type and frequency of analgesic medication is associated with frailty. The administration of analgesics in the older adult is more likely to increase the risk of adverse effects, including dizziness, falls, and fractures.

Our study has several strengths. Firstly, the study was designed as a prospective study to exclude as much as possible the influence of confounding factors on the results. Secondly, the frailty assessment scale that is easy to administer and more applicable to clinical older adults was used for better feasibility. Third, we utilized HZ, prevalent among older persons, as a study context to explore the impact of PHN on the heightened risk of frailty.

This study has inherent limitations. This study had a limited sample size and was biased by exclusively including hospitalized patients with HZ. Secondly, frailty is a dynamic and evolving process; additional follow-up is required to evaluate the correlation between PHN and frailty.

## 5 Conclusion

Older adult patients with PHN are at a higher risk of developing frailty compared to those without PHN, and PHN is a significant contributor to frailty. Consequently, it is crucial to develop scientifically effective interventions for the prevention and treatment of PHN, and future studies should explore the efficacy of different PHN management strategies in mitigating the incidence of frailty.

## Data Availability

The raw data supporting the conclusions of this article will be made available by the authors, without undue reservation.
